# Neuroprotective Effect of Taurine against Cell Death, Glial Changes, and Neuronal Loss in the Cerebellum of Rats Exposed to Chronic-Recurrent Neuroinflammation Induced by LPS

**DOI:** 10.1155/2021/7497185

**Published:** 2021-07-05

**Authors:** Samara P. Silva, Adriana M. Zago, Fabiano B. Carvalho, Lucas Germann, Gabriela de M. Colombo, Francine L. Rahmeier, Jessié M. Gutierres, Cristina R. Reschke, Margarete D. Bagatini, Charles E. Assmann, Marilda da C. Fernandes

**Affiliations:** ^1^Pathology Research Laboratory, Federal University of Health Sciences of Porto Alegre, Sarmento Leite 245, CEP 90050-170 Porto Alegre, RS, Brazil; ^2^School of Pharmacy and Biomolecular Sciences, RCSI University of Medicine and Health Sciences, Dublin, D02 YN77, Ireland; ^3^FutureNeuro, The Science Foundation Ireland Research Centre for Chronic and Rare Neurological Diseases, RCSI University of Medicine and Health Sciences, Dublin, D02 YN77, Ireland; ^4^Graduate Program in Medical Sciences, Federal University of Fronteira Sul, Chapecó, SC, Brazil; ^5^Postgraduate Program in Biological Sciences: Toxicological Biochemistry, Federal University of Santa Maria, Santa Maria, RS, Brazil

## Abstract

The present study investigated the neuroprotective effect of taurine against the deleterious effects of chronic-recurrent neuroinflammation induced by LPS in the cerebellum of rats. Adult male *Wistar* rats were treated with taurine for 28 days. Taurine was administered at a dose of 30 or 100 mg/kg, by gavage. On days 7, 14, 21, and 28, the animals received LPS (250 *μ*g/kg) intraperitoneally. The vehicle used was saline. The animals were divided into six groups: vehicle, taurine 30 mg/kg, taurine 100 mg/kg, LPS, LPS *plus* taurine 30 mg/kg, and LPS *plus* taurine 100 mg/kg. On day 29, the animals were euthanized, and the cerebellum was removed and prepared for immunofluorescence analysis using antibodies of GFAP, NeuN, CD11b, and cleaved caspase-3. LPS group showed a reduction in the immunoreactivity of GFAP in the *arbor vitae* and medullary center and of NeuN in the granular layer of the cerebellar cortex. LPS increased the immunoreactivity of CD11b in the *arbor vitae* and in the medullary center. Taurine protected against these effects induced by LPS in immunoreactivity of GFAP, NeuN, and CD11b, with the 100 mg/kg dose being the most effective. LPS induced an increase in the number of positive cleaved caspase-3 cells in the Purkinje cell layers, granular layer, *arbor vitae*, and medullary center. Taurine showed its antiapoptotic activity by reducing the cleaved caspase-3 cells in relation to the LPS group. Here, a potential neuroprotective role of taurine can be seen since this amino acid was effective in protecting the cerebellum of rats against cell death and changes in glial and neuronal cells in the face of chronic-recurrent neuroinflammation.

## 1. Introduction

Neurological and neurodegenerative diseases are devastating conditions that can affect different brain structures, including the cerebellum [1]. The cerebellum is a central brain structure deeply integrated into major loops with the cerebral cortex, brainstem, and spinal cord and is essential for the performance of smooth and accurate goal-directed movements, making postural adjustments to maintain balance and also learning new motor skills [2–4]. New evidence points to the role of the cerebellum in almost all neurological functions, including cognitive, emotional-social-psychological process, and lesions of its different parts affect each of these domains [5–7].

Neuroinflammation is considered a hallmark of brain diseases [8]. It is characterized by an increase of proinflammatory mediators and in the quantity of apoptotic neurons [9, 10] and often accompanies and/or precedes the development of pathologies such as ataxia, Parkinson's and Alzheimer's diseases [11], and epilepsy [12]. An important tool to mimic neuroinflammation *in vivo* is the administration of lipopolysaccharide (LPS), a molecule present in the outer membrane of Gram-negative bacteria. LPS causes an immediate systemic inflammatory response mainly by activating the toll-like receptor (TLR) 4, although there is also evidence of its interaction with transient r*eceptor* potential- (TRP-) like channels [13, 14]. Generally, LPS is used in order to stimulate glial cells, mainly microglia. However, astrocytes and some populations of neurons also express TLR4 receptors, becoming a target of this toxin, either by direct or indirect mechanisms [15–19]. Notably, microglial cells and macrophages may increase their activity mediated by cytokines and chemokines, such as tumor necrosis factor alpha (TNF-*α*), interleukin- (IL-) 1*β*, and nuclear factor-kappa B (NF-*κβ*) [9, 20–22].

The use of nutraceuticals has gained predominance in recent years. For decades, nutritional errors have been attributed to the onset of chronic diseases, and an adequate diet or replacement of nutrients may be the key to a good quality of life. Taurine (2-aminoethanesulfonic acid) is a free amino acid commonly found in human tissues [23–26], which can be synthesized in the body from the amino acids methionine and cysteine [27, 28]. Another source of taurine is diet since this amino acid is absorbed from foods that include meat, nuts, seafood, beans, milk, and their derivatives [26]. Taurine acts as Anti-inflammatory agent suppressing inducible nitric oxide synthase (iNOS), cyclooxygenase 2 (COX-2), and prostaglandin E2 expression and has inhibitory effects against the NF-*κβ* p65 and NF-*κ*B DNA-binding activity on exposed macrophages to the LPS [29]. In the CNS, it regulates ion channels significantly influencing neuronal activity [23–26]. It has been suggested that taurine supplementation to antiseizure drugs may be a promising approach [30].

In the CNS, the upregulation of taurine gene 1 attenuates inflammation via targeting NF-*κ*B1/p50 in a model to multiple sclerosis [31]. Taurine has the ability to neutralize the deleterious effects caused by reactive species and regular pathways of apoptosis in neurons and astrocytes, protecting them from cell death [32–34]. Furthermore, taurine effectively maintains neurogenesis in subgranular zone (SGZ) and attenuates the increase in hippocampal microgliosis and peripheral proinflammatory cytokines induced by LPS [35].

Based on this evidence, we designed a study to assess whether supplementation with taurine attenuates glial activation, neuronal death, and apoptosis in the cerebellum of rats exposed to LPS-induced chronic-recurrent neuroinflammation.

## 2. Materials and Methods

### 2.1. Chemicals

Lipopolysaccharides from *Escherichia coli* (055:B5) and taurine (TAU, ≥99%, T0625-500G) were obtained from Sigma-Aldrich Chemical Co. (St. Louis, MO, USA). All other reagents used in the experiments were of analytical grade and of highest purity.

### 2.2. Animals

Male adult *Wistar* rats (*n* = 48) weighing 300 g on average, from the local breeding colony of Universidade Federal de Ciências da Saúde de Porto Alegre (UFCSPA, Brazil), were used. All procedures were approved by the Ethics Committee of UFCSPA (Protocol 192/16, Identification code: 488/16). The animals were maintained in the Central Animal House of the UFCSPA in colony cages at an ambient temperature of 23 ± 2°C and relative humidity of 45–55% with 12 h light/dark cycles. The animals had free access to a standard rodent pellet diet and water *ad libitum*.

### 2.3. Experimental Protocol

#### 2.3.1. Treatments

**Figure 1:**
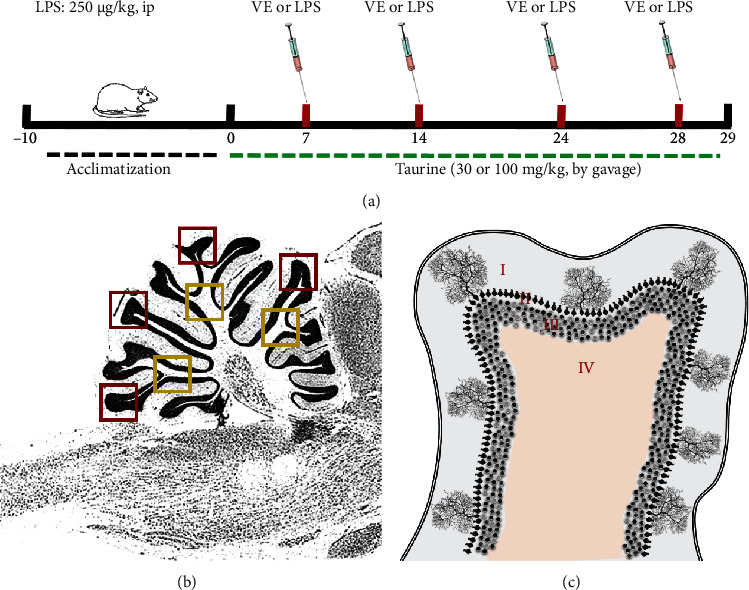
(a) Schematic illustration of experimental protocol for the taurine (TAU) at doses of 30 or 100 mg/kg and chronically exposed to lipopolysaccharide (LPS) toxin (250 *μ*g/kg, intraperitoneally) treatments of rats. First, the rats were acclimatized for 10 days. Then, the treatment with TAU occurred once a day through an intragastric tube (gavage) during days 0 until 28. The LPS toxin was administered on days 7, 14, 21, and 28. On day 29, the animals were euthanized for cerebellar acquisition. (b) Representation of the fields chosen for the acquisition of the images of the tissue sections. A total of 5 sections were used for each rat. The red squares point to the cerebellar cortex of the cerebellar leaflets. The yellow squares show the regions chosen for the *arbor vitae*. The blue squares show the areas where images of the medullary center of the cerebellum were obtained. The images were acquired in a magnification of 200x, objective lens 20x, and field area of 200,500.10 *μ*m^2^. (c) Representation of the layers of the cerebellar leaflets: I, molecular layer; II, Purkinje cell layer; III, granular layer; and IV, white matter.

Rats were treated by gavage (1 ml/kg) with taurine in the doses of 30 and 100 mg/kg body weight, previously dissolved in saline during 28 days (at 9 : 00 am). The doses were chosen based on a previous study by our research group [34]. LPS was dissolved in saline, and the selected dose was 250 *μ*g/kg as previously described [9, 36], and this toxin was administered intraperitoneally (i.p.) on days 7, 14, 21, and 28 (mimicking a chronic-recurrent neuroinflammation; [37]). A total of 4 administrations were performed. The control groups received only the vehicle (1 ml/kg of saline, i.p.). Rats were randomly distributed into six groups: vehicle, taurine 30 mg/kg (TAU30), taurine 100 mg/kg (TAU100), LPS, LPS *plus* taurine 30 mg/kg (LPS30), and LPS *plus* taurine 100 mg/kg (LPS100). Further information can be viewed in the experimental design (Figure 1(a)).

### 2.4. Preparation of Samples for Immunofluorescence Analysis

On day 29, the animals were deeply anesthetized with an i.p. injection of ketamine (80 mg/kg; Syntech) and xylazine (5 mg/kg; Syntech 2%) and transcardially perfused with saline 0.9% (during 10 min) and 4% paraformaldehyde (PFA, during 30 min) in 1% phosphate-buffered saline (PBS at pH 7.4). The cerebellums were postfixed in 4% PFA (24 hours), transferred to a solution of 30% sucrose in PBS-1% until total submersion [38]. Then, the frozen fixed cerebellums were sectioned (5 and 16 *μ*m coronal sections) using a cryostat Leica CM3050S (Leica Microsystem, German). Next, the sections were mounted on slides coated with 2% gelatin plus 0.08% chromalin (chromium and potassium sulfate, from Sigma-Aldrich, Brazil) and finally allowed to dry at room temperature during 24 hours. At last, all sections were stored at -20°C until use.

### 2.5. Immunofluorescence

Firstly, the cryosections were immersed in cold acetone (4°C, PA) for 10 minutes. After, the sections were washed in PBS twice (10 min) then blocked/permeabilized with 5% bovine serum albumin (BSA) and PBS plus 0.1% Triton X-100 for 2 hours at room temperature (RT). Sections were incubated with primary antibodies diluted in 5% BSA solution: (a) Mouse anti-CD11B (sections of 16 *μ*m, microglia marker) 1 : 500, overnight at 4°C, Abcam followed by secondary antibody (goat anti-mouse alexa fluor 488, 1 : 500, 2 hr, RT in the dark; Thermo Fischer); (b) Rabbit anti-GFAP (sections of 16 *μ*m, astrocyte marker) 1 : 800, overnight at 4°C, DAKO followed by secondary antibody (goat anti-rabbit alexa fluor 555, 1 : 1,000, 2 hr, RT in the dark; Thermo Fischer); (c) Mouse anti-NeuN (sections of 5 *μ*m, mature neuronal marker) 1 : 6,000, overnight at 4°C, MilliPore followed by secondary antibody (goat anti-mouse alexa fluor 488, 1 : 500, 2 hr, RT in the dark, Thermo Fischer); and (d) Rabbit anticleaved caspase-3 (sections of 16 *μ*m, apoptosis marker) 1 : 200, overnight at 4°C, Cell Signaling followed by secondary antibody (goat anti-rabbit alexa 555, 1 : 1,000, 2 hr RT in the dark, ThermoFischer). The 4′,6-diamidino-2-phenylindole (DAPI) solution (1 *μ*g/ml) was prepared in PBS-Tx, and an incubation was carried out for 10 minutes in the dark. Then, the sections were washed 4 times with PBS-Tx for 5 minutes each. Vecta-Shield was added over the sections which were then overlaid with a coverslip.

The images were acquired using a Leica DM6-B microscope, Leica DFC 7000-T camera, and Leica Las X software. A total of 5 sections were used for each rat (see Figure 1(b)). For each section, 4 images were acquired on the cerebellar leaflets (cerebellar cortex in the red squares and *arbor vitae* in the yellow ones) and 3 images on the medullary center (blue squares, see Figure 1(b)). The images were acquired in a magnification of 200x, objective lens 20x, and field area of 200.500,10 *μ*m^2^.

The quantification of GFAP immunoreactivity occurred in the center of the *arbor vitae* and the cerebellar leaflet. The quantification of NeuN immunoreactivity occurred in the granular layer located in the center of the cerebellar leaflets and at the edge of the medullary center. Quantification of CD11b occurred in the middle of both the *arbor vita*e and the medullary center. The positive cleaved caspase-3 cells were counted in the molecular layer of the cerebellar leaflets, in the Purkinje cells layer, in the granular layer of the cerebellar leaflets, in the white substance of *arbor vitae*, and in the medullary center. Optical density was performed using the Image Pro-Plus®.

### 2.6. Statistical Analysis

The normality analysis of the samples was performed by Kolmogorov Smirnov test. Afterwards, parametric data were analyzed using two-way analysis of variance (ANOVA) followed by the Bonferroni test when appropriate. Results were expressed as the mean ± standard error (SE). *P* values less than 0.05 (*P* < 0.05) were considered as indicative of significance.

## 3. Results and Discussion

### 3.1. Taurine Restores Immunoreactivity of GFAP in the Cerebellum of Rats Subjected to Chronic-Recurrent Neuroinflammation

**Figure 2:**
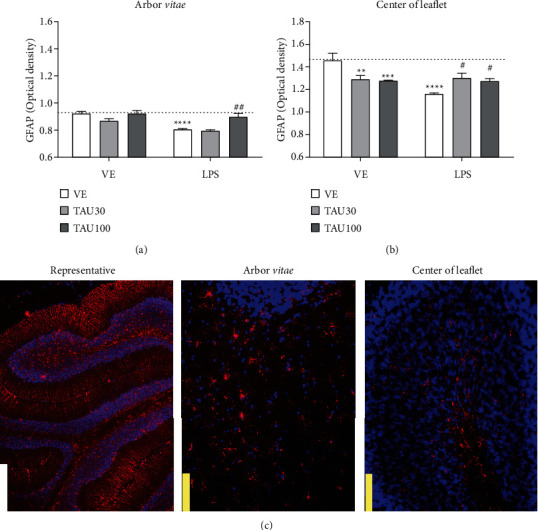
Neuroprotective effect of taurine (TAU, 30-100 mg/kg by gavage; vehicle saline) on the reduction of glial fibrillary acid protein (GFAP) in the *arbor vitae* (a) and in the center of leaflet (b) regions of rats exposed to chronic-recurrent neuroinflammation induced by the LPS toxin (250 *μ*g/kg, intraperitoneally; vehicle saline). (c) Representative image of the immunostaining obtained through the fluorescence of the selected fields (white bar: 500 *μ*m; yellow bar: 100 *μ*m). *P* < 0.05 was considered to represent a significant difference. ^∗^ denotes a significant difference compared to the vehicle group (VE). # denotes a significant difference compared to the LPS group. Two-way ANOVA followed by Bonferroni's post hoc. ^∗^ or # denotes a difference of *P* < 0.05; ^∗∗^ or ## denotes a difference of *P* < 0.01; ^∗∗∗^ or ### denotes a difference of *P* < 0.001. Data are expressed as mean ± standard error of the mean (SEM), *n* = 5-6 per group.

Figure 2 shows the immunoreactivity of the GFAP protein in the *arbor vitae* and medullary center in the cerebellum of rats submitted to chronic-recurrent neuroinflammation induced by LPS and treated with different doses of taurine.  Figure 2(a) shows a significant interaction between taurine versus LPS treatments (*F*_(2.28)_ = 3,912, *P* < 0.05) for the GFAP in the arbor vitae. LPS reduced the GFAP immunoreactivity in relation to the vehicle group (*F*_(1.28)_ = 24.37, *P* < 0.001; Figure 2(a)). Taurine 100 mg/kg protected against reduction of GFAP immunoreactivity induced by LPS (*P* < 0.01; Figure 2(a)); however, similar results were not met by taurine 30 mg/kg. In the medullary center, we verified that LPS also reduced immunoreactivity of GFAP in relation to the vehicle group (*F*_(1.26)_ = 6.678, *P* < 0.05; Figure 2(b)). Taurine reduced immunoreactivity of GFAP *per se* at doses of 30 (*P* < 0.01) and 100 mg/kg (*P* < 0.001). A significant interaction between taurine versus LPS treatments was also seen, showing that taurine protected against the reduction of GFAP induced by LPS at doses of 30 and 100 mg/kg (*F*_(2.26)_ = 8.102, *P* < 0.05, Figure 2(b)).

Astrocytes, microglia, and neurons may be affected by LPS via TLR-4 expressed on their membranes or by the proinflammatory cascade mediated by this toxin. Thus, studying these cell populations against an inflammatory condition is extremely relevant. The glial fibrillary acidic protein (GFAP) is an intermediate filament protein present in astrocytes, and its quantification is widely used to verify astrogliosis. Astrocytes have a range of control and homeostatic functions in health and disease and assume a reactive phenotype in acute central nervous system (CNS) traumas, ischemia, and in neurodegenerative diseases [39]. In acute conditions, LPS stimulates the expression of GFAP, protecting neuronal activity during inflammatory challenges [40]. On the other hand, microglia comprise between 5 and 20% of the total glial cell population, are more frequent in the grey matter, and found throughout the normal mammalian CNS [41]. The microglia are the only immune cells present in the CNS parenchyma and are thus the first responders to environmental change. Under conditions of tissue damage such as that associated with bacterial or viral infections of the CNS, microglia play a critical role in clearing debris and restoring homeostasis in the CNS [42].

Fu et al. [43] verified astrogliosis in rats exposed to LPS (2 mg/kg) over 30 days. Through this time-curve, after a single i.p. injection, there is an increase in immunoreactivity of GFAP between days 1 and 7 [43]. Acute exposures to LPS are capable of inducing astrogliosis by elevating GFAP content and immunoreactivity in the nervous system, highlighting the hippocampus [44] and cerebellum [45]. As reported by Perez-Dominguez et al. [37], until seven days after the systemic LPS challenge, astrocytes present mild cell body hypertrophy, extended cell processes, and an increased GFAP immunoreactivity in the hippocampus. However, a repeated LPS exposure did not elicit an evident astrocytic reaction suggesting a lack of persistent astrocytic response after a repeated inflammatory challenge [37]. The same effect was observed by Fu et al. [43], who reported a gradual increase in the expression of GFAP, IL-1*β*, and TNF-*α* in the hippocampus up to seven days after the administration of 2 mg/kg of LPS. After the seventh day, there were no significant differences in the GFAP expression [43]. We observed the same effect of LPS on GFAP in both regions studied, but only in the central medullary region the dose of taurine 30 mg/kg showed a protective effect. Although LPS-induced neuroinflammation has been pivotally associated with microglia activation, astrocytes play an important role in maintaining brain homeostasis and protecting surrounding neurons from damage like infective agents [46].

### 3.2. Taurine Prevents LPS-Induced Neuronal Death in the Rat Cerebellum

**Figure 3:**
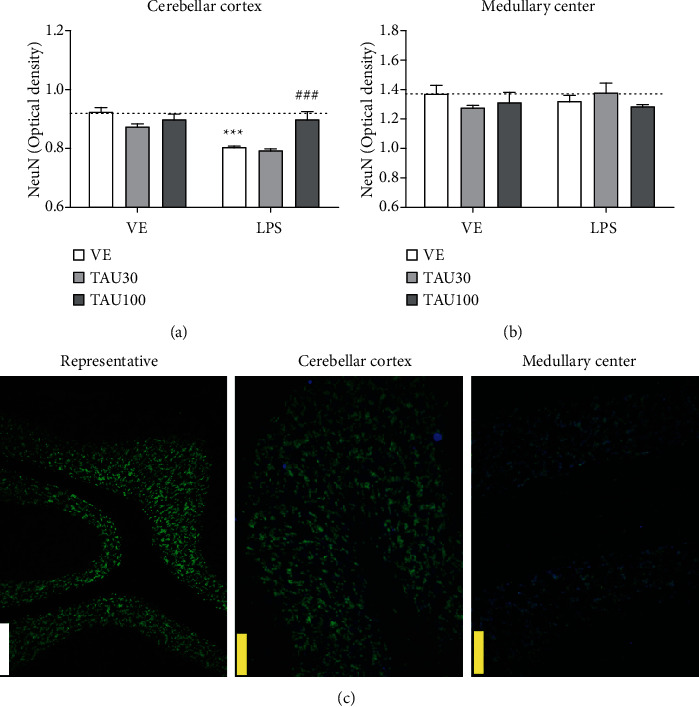
Neuroprotective effect of taurine (TAU, 30-100 mg/kg, by gavage; vehicle saline) on the reduction of immunoreactivity for the NeuN protein in the cerebellar cortex (a) and edges of the medullary center (b) regions of rats exposed to chronic-recurrent neuroinflammation induced by the LPS toxin (250 *μ*g/kg, intraperitoneally; vehicle saline). (c) Representative image of the immunostaining obtained through the fluorescence of the selected fields (white bar: 500 *μ*m; yellow bar: 100 *μ*m). *P* < 0.05 was considered to represent a significant difference. ^∗^ denotes a significant difference compared to the vehicle group (VE). # denotes a significant difference compared to the LPS group. Two-way ANOVA followed by Bonferroni's post hoc. ^∗∗∗^ or ### denotes a difference of *P* < 0.001. Data are expressed as mean ± standard error of the mean (SEM), *n* = 5-6 per group.

Figure 3 shows the immunoreactivity for the NeuN protein in the regions of the cerebellar cortex and at the edges of the medullary center of the cerebellum of rats subjected to chronic-recurrent neuroinflammation induced by LPS and treated with different doses of taurine.  Figure 3(a) shows a significant interaction between taurine versus LPS treatments (*F*_(2.26)_ = 7,539, *P* < 0.01) for the NeuN protein in the cerebellar cortex. LPS reduced the immunoreactivity of the NeuN protein in relation to the vehicle group (*F*_(1.26)_ = 26.26, *P* < 0.001; Figure 3(a)). Again, taurine 100 mg/kg, but not 30 mg/kg, protected against the reduction in immunoreactivity for NeuN induced by LPS (*P* < 0.001; Figure 3(a)). In the granular layer present at the edge of the medullary center, no significant differences were observed between treatments for the immunoreactivity of the NeuN protein (*F*_(2.26)_ = 0.2621, *P* > 0.05; Figure 3(b)). Neuronal nuclei (NeuN) is a well-recognized marker that is exclusively detected in postmitotic neurons. NeuN is distributed in the nuclei of mature neurons and has been considered a reliable marker of mature neurons in certain diseases and specific physiological states [47].

Pinato and colleagues [48] reported that intracerebroventricular LPS triggers a reduction in immunoreactivity for NeuN in the cerebral cortex, in the dentate gyrus of the hippocampus, and in the granular layer of the cerebellum of rats. The same effect is reproduced in cerebellar cell culture. In addition, the authors described that this reduction was also accompanied by an increase in positive fluoro-jade cells, which indicate a death of adult neurons by neuroinflammation [48]. Although inflammation is implicated in the progressive nature of neurodegenerative conditions, such as Alzheimer's and Parkinson's diseases [49], and also in seizure recurrence in epilepsy [19], the mechanisms are yet poorly understood. Systemic LPS administration results in rapid brain TNF-*α* increase by activating brain microglia to produce chronically elevated proinflammatory factors and culminating in delayed and progressive loss of neurons in the nervous system [50, 51].

### 3.3. Taurine Suppresses Microglial Immunoreactivity in the Cerebellum of Rats Submitted to Chronic-Recurrent Neuroinflammation Induced by LPS

**Figure 4:**
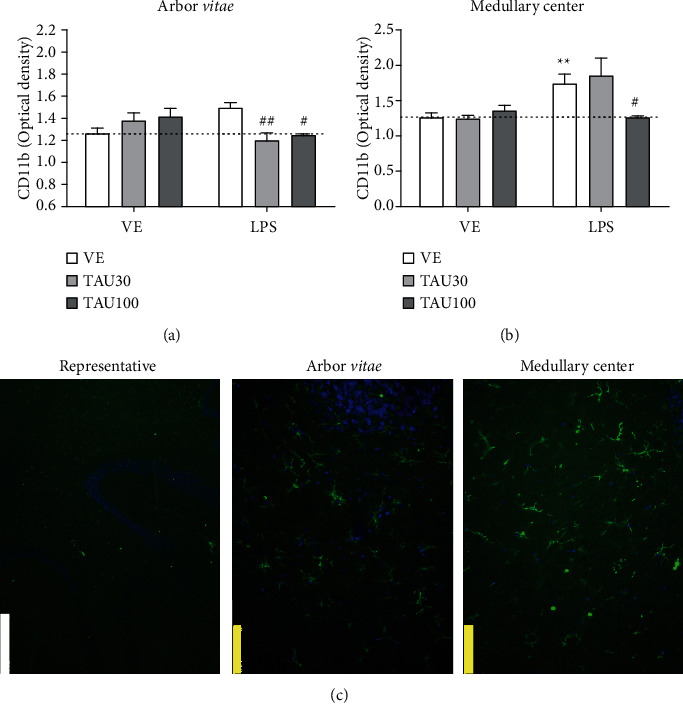
Neuroprotective effect of taurine (TAU, 30-100 mg/kg by gavage; vehicle saline) on the increase in CD11b protein in the *arbor vitae* (a) and medullary center (b) regions of rats exposed to chronic-recurrent neuroinflammation induced by the LPS toxin (250 *μ*g/kg, intraperitoneally; vehicle saline). (c) Representative image of the immunostaining obtained through the fluorescence of the selected fields (white bar: 500 *μ*m; yellow bar: 100 *μ*m). *P* < 0.05 was considered to represent a significant difference. ^∗^ denotes a significant difference compared to the vehicle group (VE). # denotes a significant difference compared to the LPS group. Two-way ANOVA followed by Bonferroni's post hoc. ^∗^ or # denotes a difference of *P* < 0.05; ^∗∗^ or ## denotes a difference of *P* < 0.01. Data are expressed as mean ± standard error of the mean (SEM), *n* = 5-6 per group.

Figure 4 shows the immunoreactivity of the CD11b protein in the *arbor vitae* and medullary center of the cerebellum of rats submitted to chronic-recurrent neuroinflammation induced by LPS and treated with different doses of taurine.  Figure 4(a) shows a significant interaction between taurine versus LPS treatments (*F*_(2.32)_ = 6.428, *P* < 0.01) for the CD11b protein in the *arbor vitae*. LPS increased the immunoreactivity of the CD11b in relation to the vehicle group (*F*_(1.32)_ = 26.26, *P* < 0.001; Figure 4(a)). Treatment with either taurine 30 or 100 mg/kg prevented the expected LPS-induced microglial activation (TAU 30 mg/kg, *P* < 0.01; TAU 100 mg/kg, *P* < 0.05, Figure 4(a)). In the medullary center, only the 100 mg/kg dose was able to protect against the increased immunoreactivity of CD11b induced by LPS (*F*_(2.24)_ = 3.777, *P* < 0.05; Figure 4(b)). Microglia, which are the resident macrophages in the brain, play an important role in the occurrence and development of neuroinflammation. Under physiological conditions, microglia mainly eliminate metabolic products and toxic materials. However, if stimulated, microglia migrate to the lesion and remove cellular debris. While microglia activation is necessary and critical for host defense, excessive or prolonged activation of microglia leads to neuronal death and an increase in proinflammatory cytokines and oxidative stress [4, 9]. LPS dose-dependently increases microglial CD11b expression and is an important marker of neuroinflammation [52]. Indeed, we verified a reduction in astrogliosis and in the population of mature neurons in the cerebellum of rats, as well as an increase in microglial immunoreactivity in the LPS group. Next, we determined whether these events can be associated with apoptotic cell death in different layers and regions of the cerebellum.

### 3.4. Taurine Reduces Cell Apoptosis in Different Layers of the Cerebellum of Rats Exposed to LPS-Induced Chronic-Recurrent Neuroinflammation

**Figure 5:**
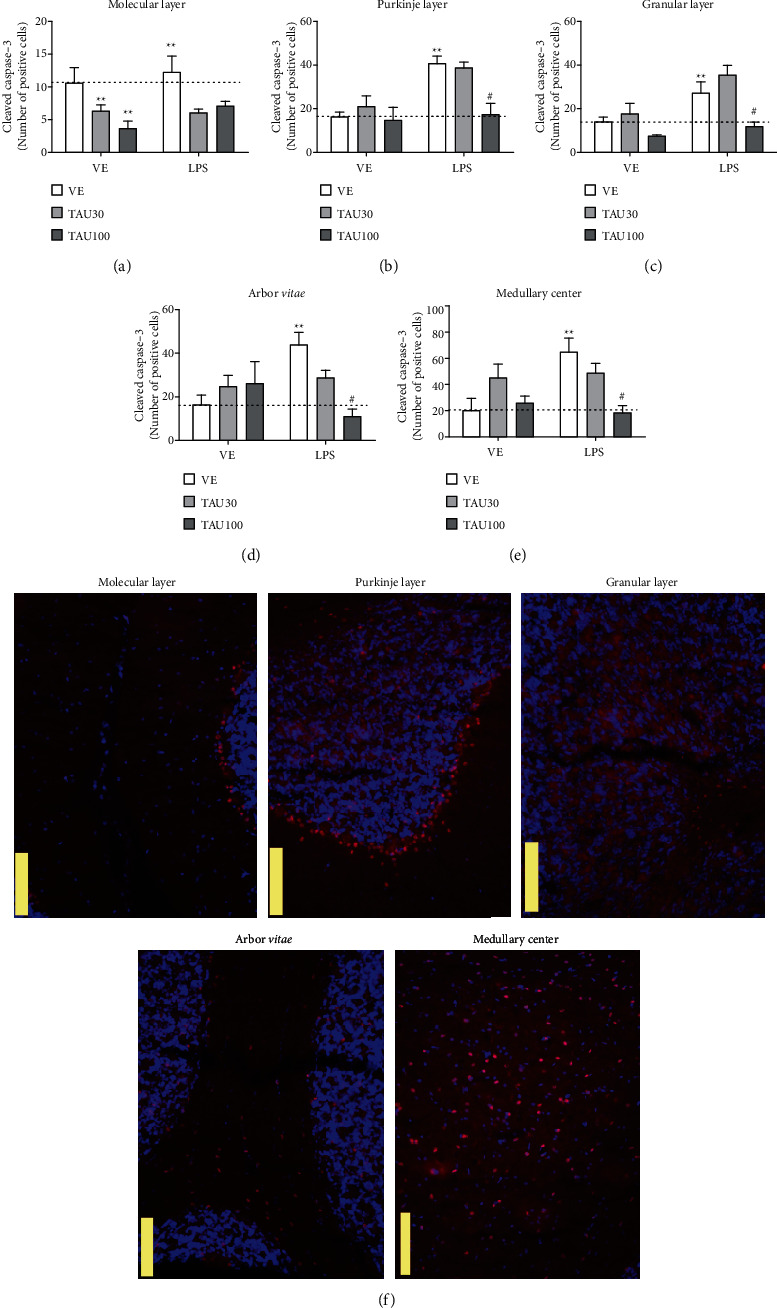
Neuroprotective effect of taurine (TAU, 30-100 mg/kg by gavage; vehicle saline) on cell apoptosis by the number of cleaved caspase-3 positive cells in the regions: (a) molecular layer, (b) Purkinje cell layer, (c) granular layer, (d) *arbor vitae*, and (e) medullary center in cerebellum of rats exposed to chronic-recurrent neuroinflammation induced by the LPS toxin (250 *μ*g/kg, intraperitoneally; vehicle saline). (f) Representative image of the immunostaining obtained through the fluorescence of the selected fields (yellow bar: 100 *μ*m). *P* < 0.05 was considered to represent a significant difference. ^∗^ denotes a significant difference compared to the vehicle group (VE). # denotes a significant difference compared to the LPS group. Two-way ANOVA followed by Bonferroni's post hoc. ^∗^ or # denotes a difference of *P* < 0.05; ^∗∗^ or ## denotes a difference of *P* < 0.01. Data are expressed as mean ± standard error of the mean (SEM), *n* = 4-5 per group.

Figure 5 shows the immunoreactivity for the cleaved caspase-3 protein in the molecular layer, granular layer, layer of Purkinje cells and white substance of the *arbor vitae*, and medullary center in the cerebellum of rats subjected to chronic-recurrent neuroinflammation induced by LPS and treated with different doses of taurine.  Figure 5(a) shows that treatment with LPS did not change the number of positive cleaved caspase-3 cells in the cerebellar molecular layer (*P* > 0.05) compared to the vehicle group. Taurine 30 and 100 mg/kg were able to reduce the number of cleaved caspase-3 cells in relation to the vehicle group (*F*_(2.20)_ = 8.21; *P* = 0.025; Figure 5(a)). There were no significant interactions between treatments (*F*_(2.20)_ = 0.6446; *P* = 0.5354; Figure 5(a)).

In the Purkinje cell layer, it was observed that LPS increased the number of positive cleaved caspase-3 cells in relation to the vehicle group (*F*_(1.20)_ = 12.21; *P* = 12.21; Figure 5(b)). The treatment with TAU 100 mg/kg, but not 30 mg/kg, protected against the increase in the number of cells in apoptosis induced by LPS (*F*_(2.20)_ = 4.985; *P* = 0.0189; Figure 5(b)). LPS also increased the number of cleaved caspase-3 cells in the granular cell layer (*F*_(1.20)_ = 12.54; *P* = 0.0019; Figure 5(c)), and treatment with TAU 100 mg/kg prevented this effect (*F*_(2.20)_ = 9.121; *P* = 0.0014; Figure 5(c)). A similar result was observed in the *arbor vitae* region where LPS triggered an increase in the number of apoptotic cells (*F*_(1.20)_ = 4.980; *P* = 0.023; Figure 5(d)), which was again prevented by TAU 100 mg/kg supplementation (*F*_(2.20)_ = 5.516; *P* = 0.0116; Figure 5(d)). Finally, apoptosis was also triggered in the medullary center of the animals in the LPS group in relation to the vehicle group (*F*_(1.20)_ = 4.099; *P* = 0.029; Figure 5(e)), which was consistently prevented by TAU 100 mg/kg treatment (*F*_(2.20)_ = 3.998; *P* = 0.0398; Figure 5(e)). Liu and colleagues showed that concentrations above 1 ng/ml of LPS induce apoptosis in microglial cell culture by analyzing TUNEL positive cells, DNA fragmentation, nuclear morphology, and quantification of cleaved caspase-3 [53]. Furthermore, intraperitoneal LPS has been shown to be able to increase not only the activity and expression of iNOS but also the number of apoptotic brain and Bax-positive cells, as well as decrease the amount of Bcl-2-positive cells [54].

It is evident that this inflammation orchestrated by the stimulation of TL4R triggered by LPS resulted in an increase in apoptosis in different regions of the cerebellum, but with no effect on the molecular layer. This chronic-recurrent process reduced the number of mature neurons and astrocytes while induced microglia activation. Several evidences have pointed out the anti-inflammatory role of taurine supplementation, and its ability has already been described to protect macrophages [55], BV2 microglial [56], and liver cells [57]. Notably, the majority of the studies focus on taurine's neuroprotective effects on the hippocampus [35, 58], and thus far, little is known about the cerebellum and its layers and regions. Further studies should be done to understand the complexity of the effects caused by chronic-recurrent inflammation on the populations that constitute the nervous system and the action mechanism of taurine as a neuroprotective agent. Here, we have verified the antiapoptotic activity of this nutraceutical agent and the ability to protect neurons and glial cells against systemic chronic-recurrent inflammation in the cerebellum.

## 4. Conclusion

Finally, taurine supplementation not only protects the cerebellum against neuronal death but also reduces microglial activation induced by the recurrent administration of LPS in the cerebellum of rats. Taurine also showed an interesting antiapoptotic activity by reducing the increase in caspase-3 cells cleaved in the molecular layer, granular layer, layer of Purkinje cells and white substance of the *arbor vitae*, and medullary center in the cerebellum. Understanding the role of taurine in the cerebellum and its regulation in several brain regions should facilitate studies on its neuroprotective mechanisms. In this regard, considering taurine as an emerging adjuvant or alternative drug for neuroprotection, further study is necessary to understand its real potential human health benefits.

## Data Availability

All results are included in the manuscript.
